# To Saratoga

**Published:** 1889-07

**Authors:** 


### To Saratoga.

The Railroad Traffic Association have agreed to furnish rates to Saratoga and return for one and one-third full fare. The regular ordinary ticket going should be bought at the starting point, taking the agent's receipt for the same, which when countersigned by the Secretary of the Association, will entitle the holder to return on the reduced rate.

Every one wishing to avail himself of this regulation must attend to it on his leaving home.

